# The Proapoptotic Influenza A Virus Protein PB1-F2 Forms a Nonselective Ion Channel

**DOI:** 10.1371/journal.pone.0011112

**Published:** 2010-06-15

**Authors:** Michael Henkel, David Mitzner, Peter Henklein, Franz-Josef Meyer-Almes, Anna Moroni, Mattia L. DiFrancesco, Leonhard M. Henkes, Michael Kreim, Stefan M. Kast, Ulrich Schubert, Gerhard Thiel

**Affiliations:** 1 Department of Botany, Technische Universität Darmstadt, Darmstadt, Germany; 2 ViroLogik GmbH, Innovation Centre for Medical Technology and Pharmaceuticals (IZMP), Erlangen, Germany; 3 Institute of Biochemistry, Humboldt University, Berlin, Germany; 4 Department of Chemistry and Biotechnology, Hochschule Darmstadt, Darmstadt, Germany; 5 Dipartimento di Biologia e IBF-CNR, Universita' degli Studi di Milano, Milano, Italy; 6 Physikalische Chemie III, Technische Universität Dortmund, Dortmund, Germany; 7 Clinical and Molecular Virology, Friedrich-Alexander-University of Erlangen-Nürnberg, Erlangen, Germany; University of Georgia, United States of America

## Abstract

**Background:**

PB1-F2 is a proapoptotic influenza A virus protein of approximately 90 amino acids in length that is located in the nucleus, cytosol and in the mitochondria membrane of infected cells. Previous studies indicated that the molecule destabilizes planar lipid bilayers and has a strong inherent tendency for multimerization. This may be correlate with its capacity to induce mitochondrial membrane depolarization.

**Methodology/Principal Findings:**

Here, we investigated whether PB1-F2 is able to form ion channels within planar lipid bilayers and microsomes. For that purpose, a set of biologically active synthetic versions of PB1-F2 (sPB1-F2) derived from the IAV isolates A/Puerto Rico/8/34(H1N1) (IAV_PR8_), from A/Brevig Mission/1/1918(H1N1) (IAV_SF2_) or the H5N1 consensus sequence (IAV_BF2_) were used. Electrical and fluorimetric measurements show that all three peptides generate in planar lipid bilayers or in liposomes, respectively, a barely selective conductance that is associated with stochastic channel type fluctuations between a closed state and at least two defined open states. Unitary channel fluctuations were also generated when a truncated protein comprising only the 37 c-terminal amino acids of sPB1-F2 was reconstituted in bilayers. Experiments were complemented by extensive molecular dynamics simulations of the truncated fragment in a lipid bilayer. The results indicate that the c-terminal region exhibits a slightly bent helical fold, which is stable and remains embedded in the bilayer for over 180 ns.

**Conclusion/Significance:**

The data support the idea that PB1-F2 is able to form protein channel pores with no appreciable selectivity in membranes and that the c-terminus is important for this function. This information could be important for drug development.

## Introduction

Influenza A Virus (IAV) is a permanent threat to humans and animals with the potential to cause disastrous pandemics which appeared periodically in the last century causing millions of fatal casualities [1], [2]. Aquatic birds are the primary reservoir of the virus. Some avian strains are able to infect other mammals or humans directly or after genetic reassortment caused by a process termed antigenic shift [3]. A major role is attributed to pigs which serve as “mixing vessel” that create avian-human reassortant strains, of pandemic potential. The recent flu pandemic caused by a influenza A virus strain of swine origin is of high interest to study the dynamics of virulence and viral spread. Though, much effort was made to unravel the precise mechanisms of IAV mediated pathogenicity in different host organisms leaving many questions to understand this complex process. Only a few years ago an 11^th^ IAV gene product was discovered. The new protein, named PB1-F2 originates from an alternative open reading frame in the PB1 polymerase gene and it is present in most human and bird flu isolates [4], [5].

Recent work has established that PB1-F2 is an important pathogenicity factor, since it has the potential to augment the generation of fatal secondary bacterial pneumonia as it was demonstrated after the expression of the 1918 IAV PB1-F2 protein in infected mice [6]. Furthermore, a single amino acid exchange from Asn-66 to Ser-66 converted an H5N1 strain of moderate pathogenicity into a highly pathogenic virus. This mutation was also found in the PB1-F2 protein of the 1918 pandemic virus isolate [7]. Interestingly, H1N1 isolates of swine origin do not express funcitonal PB1-F2, which might be a reason for the so far reported moderate pathogenicity of the new subtype variant [8].

The complete mode of action of PB1-F2 however is not yet fully understood. The protein exhibits a C-terminal mitochondrial targeting sequence (MTS) and is predominantely localized in the inner and outer mitochondrial membrane of IAV-infected or PB1-F2-transfected cells [9].

PB1-F2 is thought to initiate the intrinsic mitochondrial apoptosis pathway, presumably by depolarizing the mitochondrial membrane potential followed by subsequent Cytochrome c release [10], [11]. The mechanism by which PB1-F2 causes depolarization of the mitochondrial membrane potential is not yet resolved. This may be achieved either by an activity modulation of mitochondrial transport proteins, such as ANT3 and VDAC1 [10] and/or by a direct interaction and consequent short circuit of the mitochondrial membrane with the viral protein [12].

Structural considerations [13] and functional assays [12], [14] suggest that the biological function of PB1-F2 indeed is related to its direct interaction with membranes. The NMR structure of the *s*PB1-F2 peptide derived from IAV_PR8_ reveals a strong tendency of the protein to undergo oligomerization mediated by two distinct domains in the N- and C-termini, respectively [13]. Comparative analysis of the peptide in aqueous versus lipid environment showed that the structure of PB1-F2 is strongly determined by the surrounding solvent [13], [14]. In an aqueous environment *s*PB1-F2 is mostly present as a random coil peptide. In a lipid like environment the protein alters its structure revealing either a β-sheet or an α-helix rich structure. The NMR structure of the latter configuration reveals an α-helix at the C-terminal region between amino acid residues Ile^55^-Lys^85^. The length of this α-helix is sufficient for spanning a membrane *in trans*. Although present in different cellular sites, its potential for forming an ion channel has not been investigated thoroughly yet.

Previous electrophysiological characterizations of *s*PB1-F2 derived from the isolate IAV_PR8_ revealed that the protein can form membrane pores in planar lipid bilayers resulting in a rapid elevation of a membrane conductance with a low ion selectivity [12]. Analysis of the currents elicited by *s*PB1-F2 exhibited only random fluctuations between multiple undefined conductance levels. Current fluctuations between a defined closed and open state, which are typical for the activity of channel forming proteins, were not observed. The absence of the latter type of channel like fluctuations fostered the speculation that the protein augments membrane conductance not by protein pores but partially by lipidic pores [12]. In the present study we further tested synthetic versions of PB1-F2 proteins derived from: i) the A/Puerto Rico/8/34 (H1N1) (IAV_PR8_) strain, ii) the spanish flu isolate A/Brevig Mission/1/1918 (H1N1) (IAV_SF2_) and iii) the consensus sequence of PB1-F2 derived from 13 different bird flu virus strains (H5N1) (IAV_BF2_) in planar lipid bilayers.

Reconstitution of all three PB1-F2 molecules resulted in an elevation of membrane conductance. Close examination of the PB1-F2 evoked currents revealed in all cases typical channel like fluctuation between two dominant conductance levels. The results of these experiments underscore that PB1-F2 from distinct isolates has the ability to form non-selective protein mediated channel pores in planar lipid membranes. Since a prerequisite for forming a well-defined pore is a stable protein fold in a lipid environment in contrast to an unspecific PB1-F2-induced membrane perturbation, we tested this hypothesis by extensive molecular dynamics (MD) simulations. To this end, the NMR structure of the truncated c-terminal fragment (PDB code 2HN8, [13]) was inserted into a POPC bilayer, solvated by 150 mM KCl aqueous solution, and observed and analyzed over 180 ns. The fact that the protein remained stable in a transmembrane orientation supports the hypothesis that PB1-F2 is able to generate canonical channel activity.

## Results

**Figure 1:**
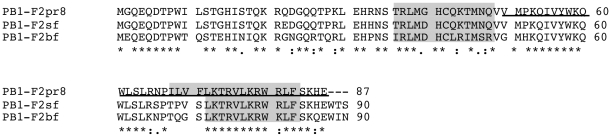
Alignment of predicted amino acid sequences of PB1-F2 proteins. The proteins from A/Puerto Rico/8/34 (H1N1) strain (PB1-F2_pr8_), the Spanish flu isolate (PB1-F2_sf_) and the bird flu virus (H5N1) (PB1-F2_bf_) have an overall identity (*) of ca 60%. The domains, which are predicted by structural prediction algorithms to have a high propensity for α-helixes are marked in gray. The truncated peptide sPB1-F2_pr8_^50–87^ is underlined.

**Figure 2:**
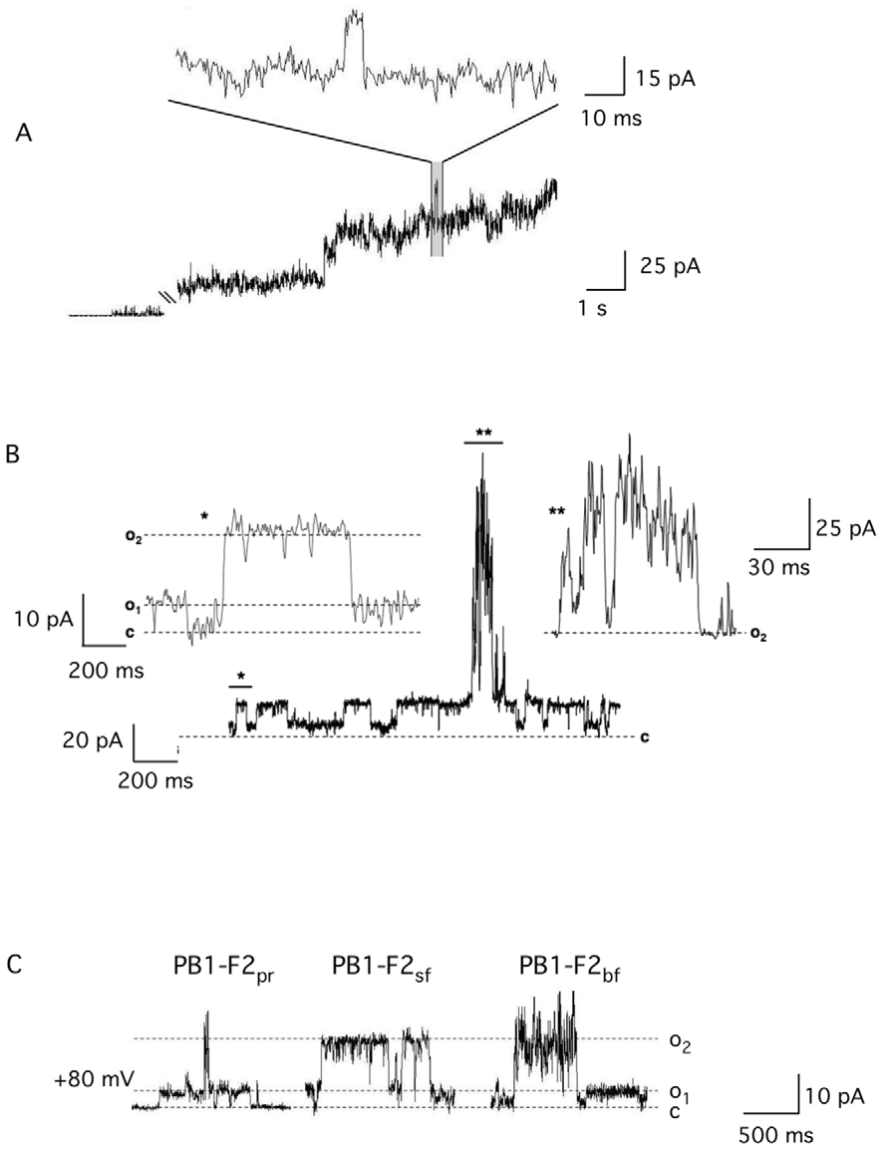
*s*PB1-F2 evoked membrane conductance. (A) Addition of *s*PB1-F2_PR8_ protein at 1 µM to the trans side of a planar lipid bilayer results in current fluctuations. In the present case only occasionally clear channel like fluctuations (see expanded trace) are resolvable on the background of many unresolved fluctuations. (B) In a majority of experiments the conductance fluctuates in a channel like manner between a closed (c) and two defined conductance levels o_1_ and o_2_. Transitions between the two conductance levels (*) are expanded in the left inset. The channel like fluctuations are occasionally interrupted by burst like events (**), which reveal also at higher magnification (inset on the right) no resolvable conductance levels.

In order to examine the mechanism by which PB1-F2 augments membrane conductance we reconstituted the synthetic protein derived from influenza A virus strain A/Puerto Rico/8/34(H1N1) (sPB1-F2_pr8_) in planar lipid bilayers. The amino acid sequence of the protein is shown in Fig. 1. After adding the protein at a final concentration of 1 µM into the bath solution (500 mM KCl, 10 mM Mops/Tris, pH 7.0) the membrane conductance increased. Fig. 2A shows exemplary currents across the bilayer in response to a voltage step from 0 mV to +80 mV before and after addition of the protein to the bath solution. In the case illustrated the viral protein evoked large current fluctuations with unresolved conductance levels; this observation is similar to that reported previously [12]. A close scrutiny of currents recorded in the presence of the sPB1-F2_pr8_ protein however shows among many unresolved fast fluctuations also typical unitary channel opening/closing events (magnification in Fig. 2A).

In the majority of experiments we observed an overall low electrical activity after addition of *s*PB1-F2_pr8_ at 1 µM. Fig. 2B shows an exemplary recording with currents across a bilayer held at +80 mV; in this case the current frequently fluctuates in an ion channel like manner between a closed state (c) and two defined conductance levels (o_1_ and o_2_); channel like activity is only occasionally interrupted by a burst of activity with no resolvable conductance levels. During the 12 min long recording the dwell time of burst like behaviour was less than 10% of the dwell time of channel type fluctuations. Worth noting is that the amplitude of the channel like fluctuations observed in this experiment (c -> o_2_) is the same as that seen in recordings with high electric activity (e.g. magnification in Fig. 2A).

**Figure 3:**
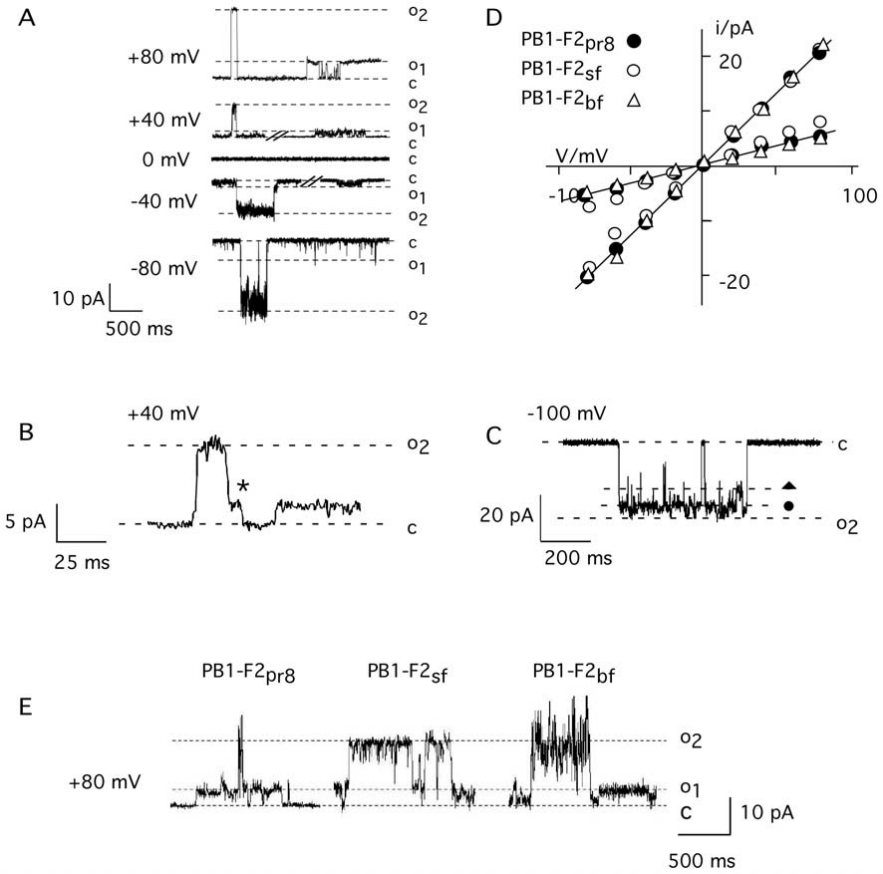
Unitary channel fluctuations and current voltage relation of sPB1-F2_pr8_ generated conductance in planar lipid bilayer. Current fluctuates between a closed (c) and two (o_1_, o_2_) conductance levels (A). Channel fluctuations recorded in symmetric 500 mM KCl in trans and cis chamber. (B) Example of channel opening in one step between closed (c) and maximal conductance (o_2_) and closing with an intermediate rest at low conductance level (*). (C) Example of intermediate closures from maximal conductance (o_2_) to sub-conductance levels, which are indicated by symbols (•,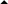). (D) I/V relation of the small (o_1_) and large current fluctuation (o_2_). Experiments performed in symmetrical 500 mM KCl. The I/V relations in D were obtained from measurements of channel conductance evoked by peptide from *s*PB1-F2_pr8_, *s*PB1-F2_sf_ and *s*PB1-F2_bf_. (E) examples of channel type current fluctuations between the closed (c) and two conductance levels (o_1_, o_2_) evoked by *s*PB1-F2 peptide from influenza Puerto Rico strain (*s*PB1-F2_pr8_), Spanish flu strain (*s*PB1-F2_sf_) and bird flu strain (*s*PB1-F2_bf_). Measurements in A-C were done in symmetric 500 mM KCl in trans and cis chamber at a voltage of +80 mV; all peptides were added to the buffer at final concentration of 1 µM.

Channel like fluctuations were observed in experiments with a protein concentration ≥20 nM. Independent on the concentration the two unitary conductance levels described in Fig. 2 prevail along with some other more rare occurring conductance levels at all voltages. Fig. 3A illustrates exemplary traces of unitary channel activity from an experiment with symmetrical 500 mM KCl. The two conductance levels are frequently achieved directly from the closed state. However the fluctuations illustrated in Fig. 3B show that the channel can also reach the closed state from the high conductance level o_2_ via an intermediate halt at the small conductance level o_1_ (B); also partial closures in which the current decreases in step like fashion from o_2_ can be observed (C). From these data we can conclude that the different conductance levels are causally related; they must be produced by the same protein or protein complex.

A plot of the two prevailing unitary current amplitudes from the closed level to o_1_ and to o_2_ as a function of voltages gives a linear current/voltage (I/V) relation for both levels of conductance in symmetrical 500 mM KCl (Fig. 3D). The conductance of the large and the small unitary opening are 250 pS and 100 pS respectively. The same results were confirmed in 4 other reconstitution experiments using protein from two different preparations.

To test whether the channel forming activity is unique to sPB1-F2_pr8_ the same experiments were repeated with synthetic peptide analogs to PB1-F2 from the ‘Spanish flu’ virus (sPB1-F2_sf_) and the bird flu virus (H5N1) (*s*PB1-F2_bf_). An alignment of the three protein sequences shows that they are 60% identical (Fig. 1). Direct comparison however also reveals deviations in the amino acid sequence throughout the protein; furthermore PB1-F2_bf_ is at the c-terminus 3 amino acids shorter than the two analogs.

Fig. 3E shows exemplary current traces at +80 mV from the three different peptides. The data reveal that all three peptides generate distinct channel fluctuations. In all cases the two dominant conductance levels o_1_ and o_2_ are again observable. The I/V curves obtained for *s*PB1-F2_sf_ and *s*PB1-F2_bf_ are indistinguishable from *s*PB1-F2_pr_ (Fig. 3D).

**Figure 4:**
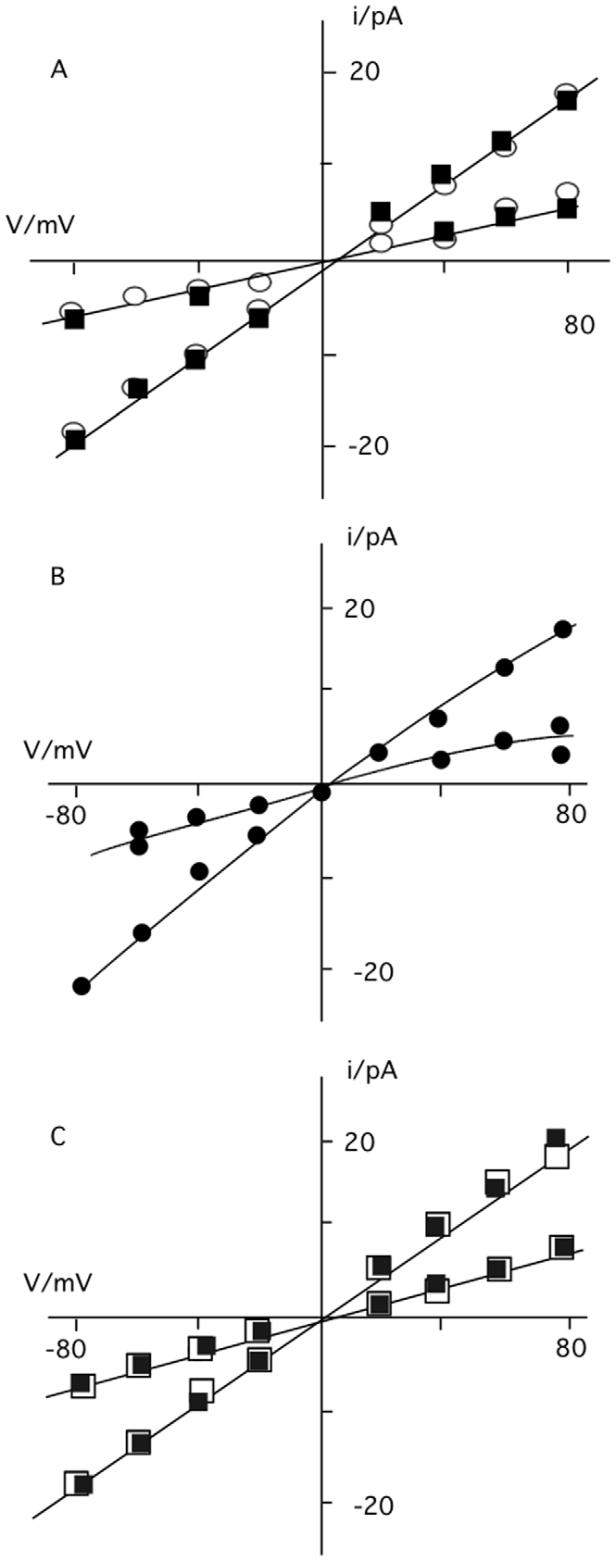
I/V relation of the small (o_1_) and large (o_2_) *s*PB1-F2 generated current fluctuation. (A) Unitary currents were recorded in bilayer with 500 mM KCl on *trans* side and 500 mM NaCl on trans (open circles) or with 500 mM KCl on cis and 500 mM K-gluconate on trans (filled squares). (B) I/V relation obtained with 500 mM KCl on trans side and 50 mM KCl on cis side. (C) I/V relation obtained with 500 mM KCl on cis and 500 mM CaCl_2_ on trans side. Currents were elicited upon adding *s*PB1-F2_pr8_ (in A-C) and sPB1-F2_sf_ (in C) to trans side.

To test the selectivity of the *s*PB1-F2_pr8_ generated channel for different ions the experiments were repeated under non-symmetrical conditions. The I/V relations obtained in experiments in which KCl in the cis chamber was replaced by either NaCl or by K-gluconate were similar to those obtained with symmetrical KCl (Fig. 4A). This means that the channel has no apparent selectivity; it neither discriminates appreciably between K^+^ and Na^+^ nor between Cl^-^ and gluconate.

Further experiments were performed with a 10-fold concentration gradient of KCl across the bilayer. The current voltage relation obtained for *s*PB1-F2_pr8_ under these non-symmetrical conditions reverses close to 0 mV at ca +5 mV. This shift in reversal voltage is real because under non-symmetrical conditions we were able to measure a negative current at 0 mV. The result of this experiment implies that the channel transports anions slightly better than cations.

In further experiments we tested whether the viral protein also transports divalent ions. For these experiments *s*PB1-F2_sf_ or *s*PB1-F2_pr8_ were reconstituted in a planar lipid bilayer with 500 mM KCl on the cis and 500 mM CaCl_2_ on the trans side. Also in these experiments we were able to detect distinct current fluctuations at positive and negative voltages (Fig. 4C and inset). The results of these experiments show that the *s*PB1-F2 generated conductance is also permeable to Ca^2+^.

**Figure 5:**
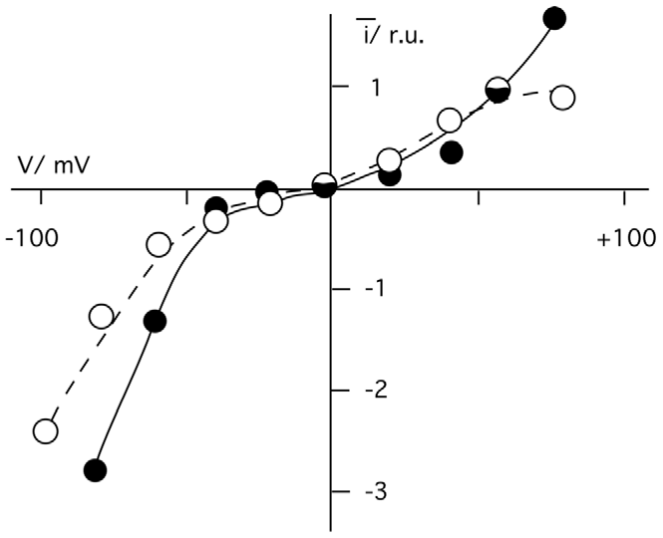
Mean current generated by *s*PB1-F2_pr8_ (filled circles) or *s*PB1-F2_sf_ (open circles) as a function of voltage. Channel activity was recorded in symmetrical 500 mM KCl. Peptide was added at 1 µM on trans side. The current responses from n = 12, 3 sec long voltage scans at the respective test voltages were averaged.

During long observations it occurred that current fluctuations were more frequent at more extreme voltages than at moderate voltages. This suggested a voltage dependency of the *s*PB1-F2 generated conductance. In order to quantify the channel activity as a function of voltage we estimated the mean current generated by sPB1-F2_pr8_ in a bilayer from n = 25 voltage steps of 3 s duration from 0 mV to test voltages between +80 mV and -80 mV. The mean steady-state currents from 12 experiments recorded in symmetrical 500 mM KCl are plotted in Fig. 5 as a function of voltage. They show a low activity at voltages around 0 mV. Towards both voltage extremes channel activity increases in a quasi-exponential fashion. Similar results were obtained with sPB1-F2_sf_; also this protein exhibits a voltage dependent I/V relation of the mean current (Fig. 5). The results of this analysis show that channel activity is irrespective of the viral origin of the sPB1-F2 protein favoured by membrane voltage. It is not possible to say whether the proteins insert into the bilayer with respect to its orientation randomly or with a bias for one side over the other. Without this information it is not yet possible to discriminate whether the channel has an inverse bell shaped open probability or whether they function as a rectifier.

**Figure 6:**
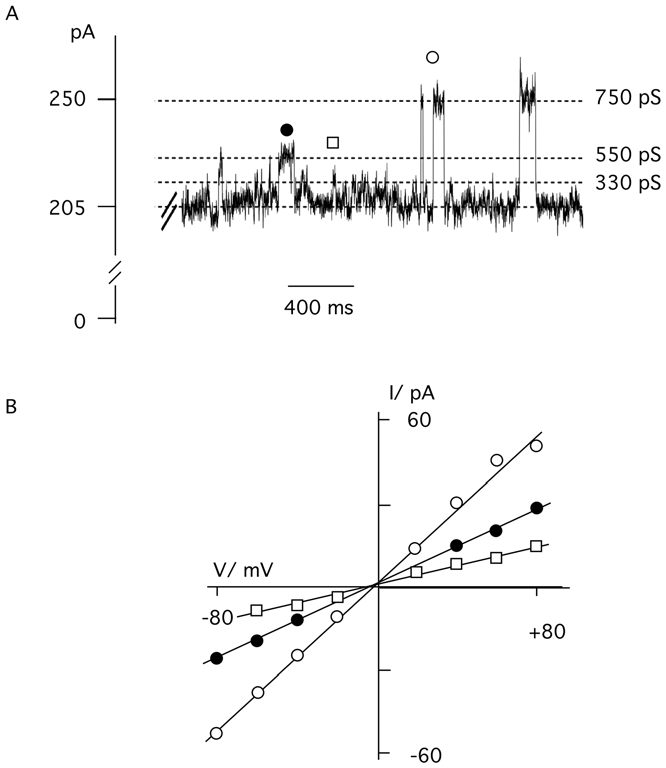
The truncated peptide *s*PB1-F2_pr8_^50–87^ generates an elevated membrane conductance. A: Addition of *s*PB1-F2_pr8_^50–87^ protein to the trans side of a planar lipid bilayer evokes large currents across the membrane. On top of many unresolved current fluctuations typical channel like opening and closing events are resolvable. In the example recorded at +80 mV three distinct current levels (indicated y different symbols) can be identified. B: a plot of similar resolvable current levels as a function of voltage reveals three linear I/V relations with 330, 550 and 750 pS. The symbols in A cross reference with symbols in B. Measurements were done in symmetric 500 mM KCl with 200 nM peptide concentration.

The NMR structure obtained for sPB1-F2_pr8_ implies a c-terminal α-helix, which is long enough to span a membrane [13]. In order to test whether this domain alone is sufficient for generating channel activity we produced a synthetic peptide, which is equivalent to the aa 50–87 of the full-length protein of PB1-F2_pr8_ [15]. The peptide (sPB1-F2_pr8_^50–87^) was tested in planar lipid bilayer in the same way as the full-length protein. Fig. 6 shows the result of a typical experiment with a symmetrical solution of 500 mM KCl; the membrane was clamped to +80 mV. Addition of the truncated peptide at a concentration of >200 nM to the trans chamber generated a large conductance in the bilayer. The current generated by sPB1-F2_pr8_^50–87^ peptide showed mostly unresolved current fluctuations. But like in the experiment of Fig. 2A it was also in these experiments possible to resolve channel like fluctuations between defined conductance levels (Fig. 6). A plot of the resolvable channel like fluctuations reveals three distinct conductance levels with 330, 550 and 750 pS. These conductance levels are not identical to those recoded with the full-length protein. Hence the putative α-helical part of the *s*PB1-F2 protein is sufficient to increase membrane conductance; it can even generate channel like activity. The difference in the resolvable conductance levels obtained with the full length compared to the truncated protein implies that the remaining n-terminal part of the protein has an influence on channel formation and unitary conductance.

### Fluorescence assay

**Figure 7:**
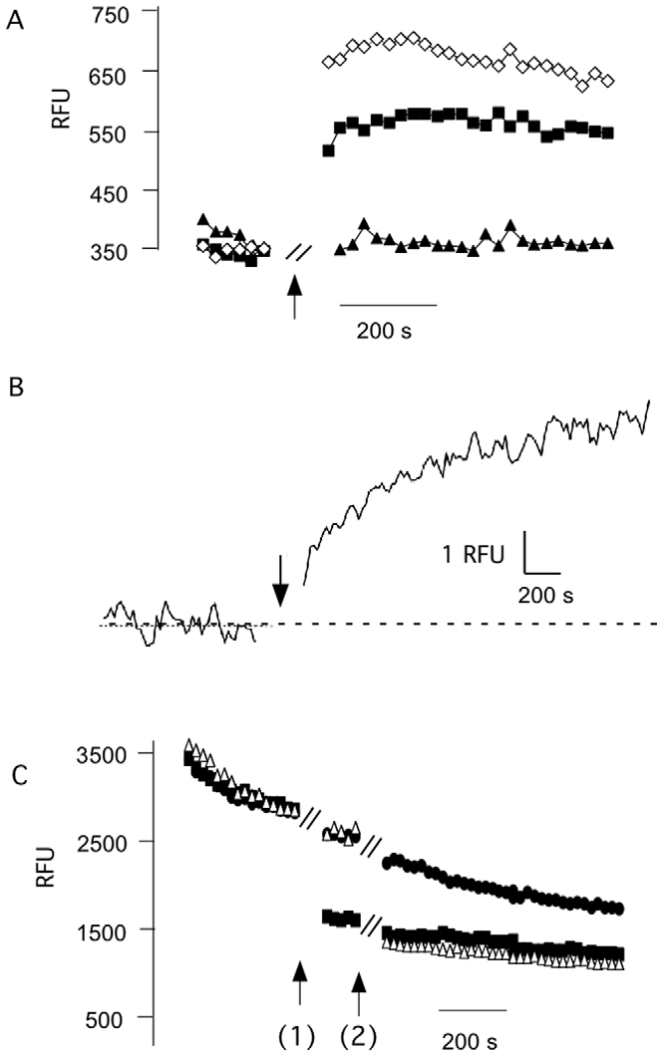
*s*PB1-F2 generates Ca^2+^ and anion fluxes into liposomes. (A) Fluorescence of liposomes with Ca^2+^ sensitive dye Fluo3 was recorded before and after adding (at arrow) ionophore Valinomycin (triangle), sPB1-F2_pr8_ alone (filled squares) or together with Valinomycin (open squares). Peptide and ionophore were added during the time gap of ca. 1 min indicated in the graph. The presence of the peptide results in an increase in fluorescence indicating an influx of Ca^2+^ into the liposomes. The ionophore enhances Ca^2+^ influx because it prevents building up of a charge, which hinders net Ca^2+^ influx. (B) Fluorescence of liposomes filled with Ca^2+^ sensitive dye Fluo-3 before and after addition (at arrow) of 1 µM peptide to incubation medium. The truncated peptide sPB1-F2_pr8_^50–87^ results in a fast rise in Fluo3 fluorescence. (C) Fluorescence of liposomes filled with anion sensitive dye lucigenin was measured before and after adding of anion specific ionophore TBT (filled squares, added at arrow 1), sPB1-F2_pr8_ (open triangle, arrow 2). The control was left untreated (filled circles); the stepwise drop of the control signal is due to an unspecific drift of the signal. Both ionophore and sPB1-F2_pr8_ generate a strong quenching of the lucigenin fluorescence well beyond the control indicating an influx of anions. Peptide and ionophore were added during the time gap of ca. 1 min indicated in the graph.

The electrophysiological data imply that the *s*PB1-F2 protein generates an unspecific conductance including permeation by Ca^2+^ and Cl^−^. This offers the possibility to examine the transport properties of PB1-F2 also in an independent assay by fluorescence spectroscopy. To measure PB1-F2 generated Ca^2+^ fluxes we loaded liposomes with the Ca^2+^ sensitive dye Fluo-3. The fluorescence intensity of the dye remained constant under control conditions. Also addition of the K^+^ selective ionophore Valinomycin had no appreciable effect on the Fluo-3 fluorescence. In contrast, addition of 1 µM *s*PB1-F2_pr8_ resulted in a rise in Fluo-3 fluorescence indicating a net influx of Ca^2+^ into the liposomes (Fig. 7). The sPB1-F2_pr8_ generated fluorescence signal could be increased by adding the peptide together with Valinomycin (Fig. 7). This enhancing effect of the ionophore on Ca^2+^ influx into the liposomes is expected because a K^+^ efflux via Valinomycin provides a charge balance for Ca^2+^ influx. In this case the membrane of the liposomes is not building up a charge, which would hinder net Ca^2+^ influx.

With a similar assay we also tested whether the truncated peptide sPB1-F2_pr8_^50–87^ is able to generate a Ca^2+^ conductance. For this purpose we loaded liposomes with Fluo-3 and measured the respective fluorescence before and after adding sPB1-F2_pr8_^50–87^ peptide to the incubation buffer. The exemplary data in Fig. 7B show that the fluorescence signal was constant before addition of the peptide. The addition of sPB1-F2_pr8_^50–87^ at a final concentration of 1 µM resulted in a quasi-immediate rise in Fluo-3 fluorescence reporting the influx of Ca^2+^ into the liposomes. The increase of relative fluorescence units (RFU) was 30%. The same response with a mean rise of 28±5% in Fluo-3 fluorescence was observed in 3 other experiments.

In further experiments we also examined the ability of *s*PB1-F2_pr8_ to stimulate Cl^-^ fluxes. In this case the liposomes were loaded with the anion sensitive dye lucigenin. Other than with Fluo-3 we observed in this type of experiments a non-specific drift of the fluorescence signal. But in spite of this shortcoming also this method could be used to demonstrate PB1-F2 generated anion fluxes. Fig. 7C shows that addition of the anion specific ionophore TBT resulted in a strong quenching of the lucigenin fluorescence indicating the sensitivity of the system. A similar drop in fluorescence was obtained upon addition of *s*PB1-F2_pr8_ at a concentration of 1 µM.

Collectively, the results of these experiments confirm the electrophysiological data in that the sPB1-F2 protein is able to generate a conductance without appreciable selectivity in lipid bilayers, which is able to conduct anions and divalent cations. The c-terminal domain of the protein with its putative α-helical structure seems to be sufficient for generating this conductance.

### MD simulation

**Figure 8:**
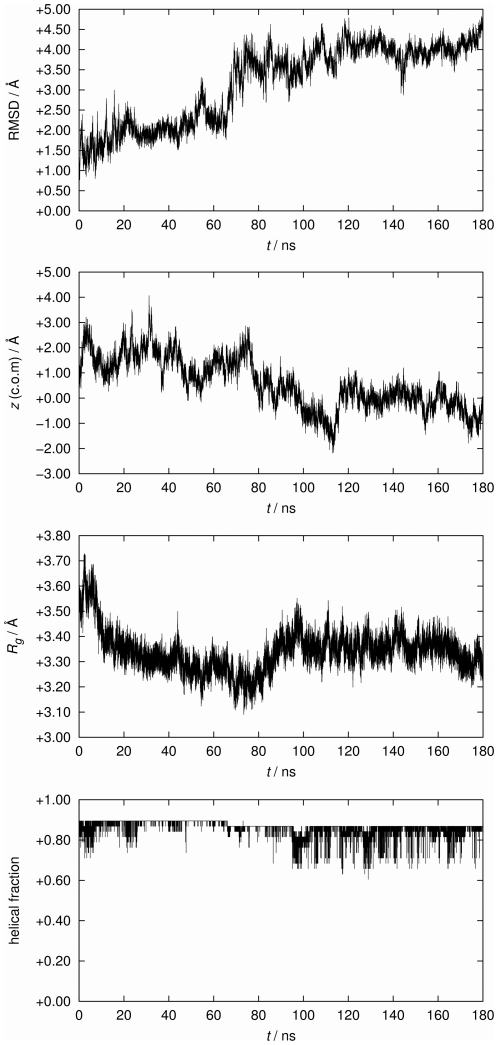
Dependence of various measures for protein stability over the simulation time after removal of the center-of-mass (c.o.m.) constraint (set to 0 ns). From top to bottom: Root mean square deviation (RMSD) of the protein backbone, *z* coordinate (membrane normal) of the c.o.m. of the protein (corrected by removing the total membrane drift), the protein's radius of gyration (*R_g_*), and the helical fraction recognized for the fold.

The electrical data imply that PB1-F2 is able to form with its c-terminus ion channels in membranes. A prerequisite for such channel formation is an insertion and a lasting transmembrane orientation of the c-terminus. To test the latter we examined the behavior of the peptide in its proposed α-helical form in a lipid environment at zero transmembrane voltage by molecular dynamics (MD) simulations. After a period with fixed center of mass of the c-terminal truncated fragment sPB1-F2_pr8_^50–87^ we observed the stability of the truncated protein by a number of quantitative measures over the last 180 ns after removal of the center-of-mass (c.o.m.) constraint. This constraint has been applied during pre-equilibration in order to fix the protein within the membrane. Quantitative results are shown in Fig. 8. All quantities indicate a remarkable overall stability of the protein fold and of the transmembrane insertion geometry. Apparently, the protein structure fluctuates within expected margins, but there is no indication for a systematic long-term trend to escape from the bilayer or to switch to a different backbone fold.

**Figure 9:**
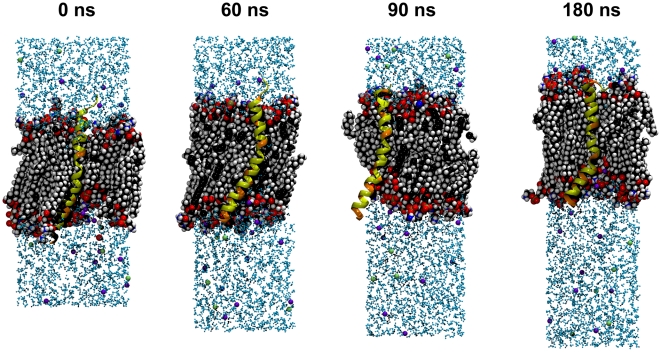
Snapshots of the simulation system after removal of the center-of-mass constraint (set to 0 ns). The protein is shown in cartoon representation with the location of positively charged amino acids marked by orange color. Potassium ions are shown in green, chloride ions in blue. The c-terminus is located on the bottom side.

**Figure 10:**
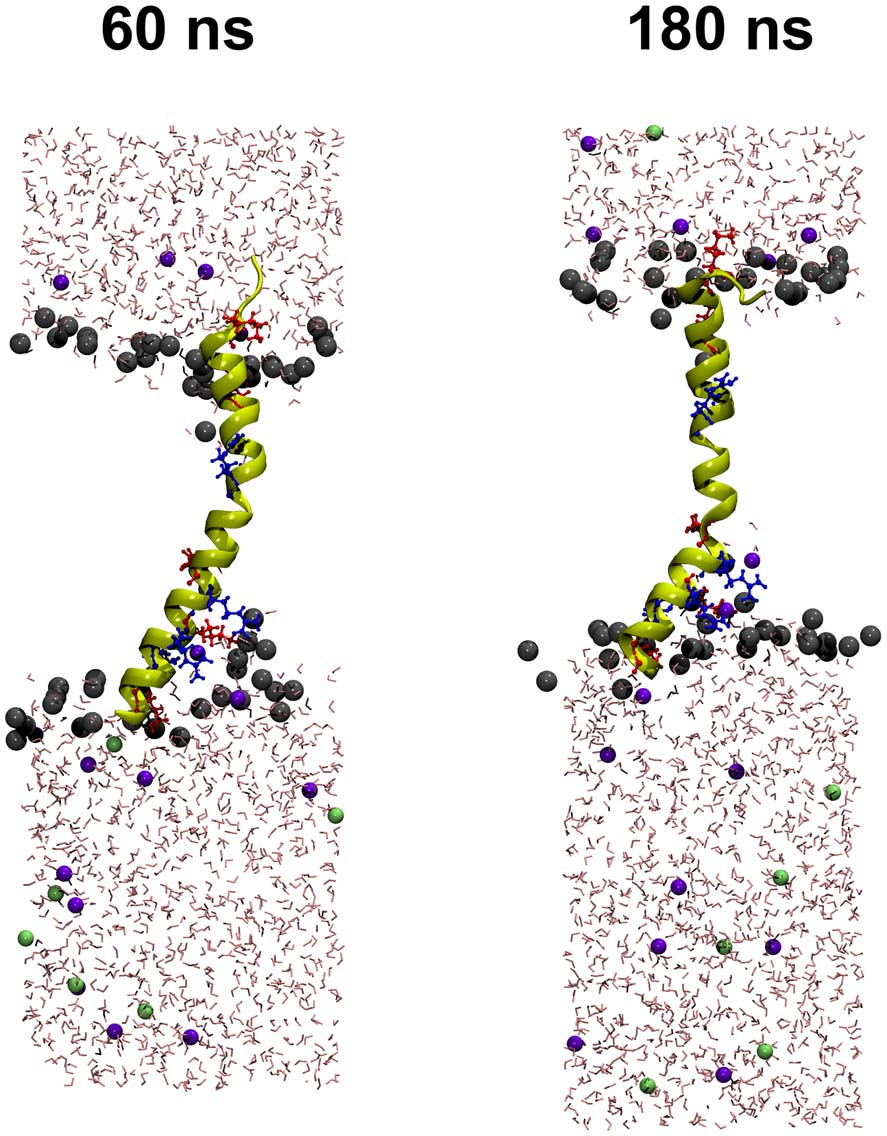
Snapshots of the simulation system after removal of the center-of-mass constraint (set to 0 ns). The protein is shown in cartoon representation with explicit depiction of positively charged residues (arginine: blue, lysine: red). Lipid molecules have been removed except for the head groups that are depicted as grey spheres. Potassium ions are shown in green, chloride ions in blue. The c-terminus is located on the bottom side.

While particularly the helical characteristic is preserved, we note a tendency to bend slightly in comparison with the NMR structure, most notable at around 70 ns where a slight step in the observables is visible. This process started shortly after removal of the c.o.m. constraint and is visualized in Fig. 9 (note that the simulation box shrinks laterally even after long time, indicating that large time scales are required for reaching an equilibrium ensemble). Bending is not unexpected since Pro^67^ is localized near the centre of the peptide. Interestingly, the Pro^67^ appears to be conserved within 263 bird flu sequences and all 20 of the H1N1 full length sequences analysed by us so far (data not shown). However, the presence of a lipid environment apparently hinders the formation of a kink. The origin of this behavior is related to the presence of a large number of positively charged amino acids (all kept in the standard protonation state during the simulation) as illustrated in Fig. 10. Arginine and lysine residues tend to “snorkel” into the direction of the membrane/solvent interface [16]. Close to the c-terminus (bottom side in Figs. 9 and 10) positive charges are clustered which leads to a net tendency to drag the protein in the direction of the c-terminus. In the presence of the c.o.m. constraint, this force is neutralized, whereas, in the absence of the constraint, the smaller number of positive charges on the n-terminal side is not sufficient to counteract fully. As a consequence, stabilization of the n-terminal side in the interface decreases such that the influence of proline becomes more important which leads to the observed bending.

It appears that the c-terminal cluster of positive charges leads to an accumulation of water and anions, which penetrate the lipid head groups. There is no indication of a persistent poration effect, however, which could be responsible for the measured channel currents. This observation requires further investigation in future work considering oligomers of the PB1-F2 peptide.

## Discussion

Early studies described PB1-F2 as a proapoptotic protein, which was assumed to remove host immune cells responding to IAV infection [4], [11]. The molecular mechanisms of PB1-F2 induced apoptosis is still not totally understood, though recent results confirmed that its proapoptotic function is cell specific and related to PKC activation after infection of primary human monocytes [17]. However, since the protein is localized predominantly in the mitochondria of transfected or infected cells and found to alter the mitochondrial morphology [4] it was hypothesized that apoptosis induction occurs due to direct interaction with subunits of the mitochondrial PTPC or destabilisation of the mitochondrial membrane by pore formation [10].

In previous experiments it has been demonstrated that PB1-F2 causes an elevation of membrane leakage [14] and increases the electrical conductance of membranes [12]. However, the currents elicited by the PB1-F2 protein were not showing defined conductance levels and channel like fluctuations. The results of these experiments therefore fostered the hypothesis that the PB1-F2 protein is not forming canonical protein mediated transmembrane pores. It was speculated that the protein may destabilize the lipid bilayer due to the formation of lipidic pores [12]. The question of how this protein destabilizes the mitochondrial membrane potential, by channel like or lipidic pore formation, is still under investigation.

The main observation of the present study is that the PB1-F2 protein is able to generate distinct opening/closing events between defined conductance levels; these events are typical for ion channel proteins [18]. Thus, it can be concluded that PB1-F2 can short circuit the mitochondria by inserting channel forming proteins into the organelle membrane. This finding might explain the property of PB1-F2 to disrupt the reticulotubular mitochondrial organization and to dissipate the inner membrane potential [4], [10]. A similar strategy for inducing apoptosis is known from a number of other viruses [19]. In the case of PB1-F2 canonical channel activity is observed with the full-length protein but also with a truncated protein comprising only the putative c-terminal α-helix (from amino acid residues Ile^55^-Lys^85^). This together with the results from MD simulations, which show that the truncated fragment is stable in the membrane, suggests that the c-terminus is part of the channel forming domain in the protein. But since the truncated and the full-length protein evoke different unitary conductances we must nonetheless assume that also the n-terminal part of the protein is involved in channel formation. Indeed the simulations show that the monomer alone leads to membrane denting, but is apparently incapable of inducing ion permeation; we did not find evidence for sufficient destabilization of the membrane to allow for passive ion translocation. Although we made no attempt to model a multi-protein complex, it is very likely that such a complex is necessary to facilitate ion transport. This hypothesis is supported by the finding that PB1-F2 has indeed the tendency to form oligomers. The latter involves distinct domains not only in the the C- but also in the N terminus [13] in line with the hypothesis, that both termini are important for the complete channel activity.

Our MD simulations imply that the c-terminus of PB1-F2 can maintain a transmembrane orientation and that this orientation is favoured by positive amino acids at the membrane/water interfaces. The data however do not yet explain how a peptide with that many charges is able to insert into the membrane. In this context it is interesting to note that also many other short, highly cationic and hydrophilic peptides, including the voltage sensor of K^+^ channels, are capable of inserting and in some cases even passing membranes [20]–[22]. The unusual behavior of these proteins can be explained by an interaction of the membrane with the distinct peptide structure; the charged amino acids in these membrane-inserting cationic peptides have to be evaluated in the overall context and their specific position in the entire peptide [20]. The structural and charge density features of the PB1-F2 protein are apparently similar to those of cationic peptides, which are able to insert spontaneously into membranes. Due to the large net charge it is very likely that the voltage dependency of the PB1-F2 conductance is related to a voltage-induced change of the positioning and orientation within the membrane. This issue needs further scrutiny by MD simulations employing an external transmembrane potential.

Both experimental and theoretical data support the view that channel activity is achieved by protein pores, which are formed by complex formation of the PB1-F2 protein. The importance of the c-terminus for channel activity is consistent with the finding that similar channel fluctuations are observed here with all three PB1-F2 analogs. The three proteins are variable in length with an overall identity of ca. 60%. Important in the context of structure/function correlates is that all three analogs contain a similar c-terminal domain with a putative transmembrane α-helix. Interestingly, all human H1N1 isolates circulating since 1950 were reported to code for a truncated PB1-F2 of 57 amino acids [5] lacking the c-terminal domain. Moreover, the current H1N1 swine flu isolates are not able to express any form of PB1-F2 since multiple Stop-codon mutations only allow the expression of the first 11 amino acids. This is consistent with initial findings that expression of PB1-F2 is species dependent and often interrupted in swine isolates [4]. Because of the missing c-terminal α-helix these proteins are therefore most likely unable to generate channel function.

The fact that the PB1-F2 peptides derived from different isolates were all able to generate similar channel like activity in planar lipid biliayers implies that the different PB1-F2 variants have the same potential of ion channel conductance within the mitochondria of infected cells. We can only speculate about the reasons why this channel type activity was recorded here but not in a previous study [12]. It is possible that the fusion efficiency of the peptides was in the present study lower than in a previous study. A lower number of proteins in the bilayer however can be beneficial for the resolution of single channel activity because individual events are not masked by the simultaneous activity of many channels. Also in the case of other pore forming peptides step like channel fluctuations can only be observed when the number of for proteins inserted into the bilayer is low [20], [23].

The analysis of PB1-F2 evoked unitary channel currents in buffers with different ion compositions reveals that the channel has two main conductance levels and both are non-selective; they transport cations as well as anions with a marginal preference for anions. The low selectivity of the channel and in particular the permeability to gluconate implies a very simple and wide channel pore. The tendency of PB1-F2 to form oligomers [13] presumably results in a self-assembly of multiple monomers in membranes with a central water filled pore. Similar channel pores with low selectivity can also be formed by aggregation of small membrane proteins from other viruses [24]–[26] or by the aforementioned alamethicin [22].

One particular feature of the PB1-F2 induced channel activity is the occurrence of at least two defined conductance levels, which can be achieved directly from the closed state. This is not unique to PB1-F2 but can also be observed with other small channel forming membrane proteins such phospholamban [27]. Also the antibiotic peptide alamethicin generates multiple unitary current levels whose conductances obey a geometrical progression; each transition is thought to result from the uptake or release of an individual monomer within the conducting bundle [28]. This explanation however does not hold true for the PB1-F2 generated conductances because inspections of the current traces also revealed intermediate closures from the high conductance level to lower levels (see Fig. 2C). Such behaviour cannot be explained by dynamic uptake or release of individual monomers from a conducting bundle. We must hence conclude that the low conductance level is a real sub-state of a channel protein build with a fixed number of monomers.

Recent data stress that PB1-F2 has the potential to modulate IAV virulence. A single amino acid exchange in the c-terminal region of the protein from Asn66 to Ser66 is sufficient to convert a virus of moderate pathogenicity into a highly pathogenic one [7]. It was also fund that the expression of the 1918 IAV PB1-F2 in mice enhanced secondary bacterial pneumonia [6]. This finding is important because most IAV related deaths are due to bacterial super-infection [6]. The present data show that the three PB1-F2 peptides from IAV of different pahtogenic potential have in spite of their sequence differences the same ability to generate ion channel activity. Hence the difference in biological function between the three PB1-F2 proteins must be due to other properties such as mitochondrial targeting or association with other proteins. Notably, the two PB1-F2 proteins, which were derived from the PR8 isolate and from the Spanish flu isolate, differ in amino acid 66, which is a Ser in IAV_SF2_ and an Arg in IAV_PR8_ (Fig. 1); this mutation, which accounts for the difference in pathogenicity [7], is within the mitochondrial targeting sequence [9].

## Methods

### Reconstitution and Electrophysiology

Experiments with planar lipid bilayers were performed as described by Schrempf et al. [29] by the Montal-Müller technique [30] with a 0.4 mg/ml solution of α-phosphatidylcholine (type IV-S> = 30% TLC; Sigma-Aldrich (Steinheim, Germany)) in n-decan (Carl Roth, Karlsruhe, Germany). sPB1-F2 was synthesized as described elsewhere [15], [31]. The measurements were done in buffer containing 500 mM KCl, 10 mM Mops/Tris pH 7. The Ag/AgCl electrode of in the trans compartment was directly connected to the head stage of a current amplifier (EPC 7, List, Darmstadt, Germany). In order to prevent surface-potential-effects both electrodes were connected with the bath solution via an agar bridge (2% agarose in 2 M KCl). Currents were recorded and stored by an analog/digital-converter (LIH 1600, HEKA electronics, Germany) at 4 KHz after low pass filtering at 1 kHz. Data analysis were performed by Patchmaster-Software (HEKA electronics) and Igor Pro (WaveMetrics, Oregon, USA). Before adding the protein to the trans chamber the bilayer conductance was recorded for some time in order to exclude artefacts from contaminations. Only perfectly silent bilayers were used for reconstitution of PB1-F2 proteins.

### Preparation of liposomes for the fluorescence assay

Lipid mixtures (100 mg/ml, S IV lipid, Sigma) were dried from CHCl3-methanol (1/1) solution under vacuum. Dried lipids were re-suspended in buffer (100 mM KCl, 10 mM Mops, titrated with Tris to pH 7.0) by vortexing; after addition of 20 µM fluorescence dye (Fluo-3, Invitrogen) liposomes were exposed to three freeze-thaw cycles. During thawing periods the liposomes were kept in an ultrasonic bath for 5 minutes. This procedure resulted in the loading of liposomes with dye. Finally the dye was removed from the external buffer by precipitation; 1ml of the dispersion was therefore washed on sephadex® -columns (Sigma Aldrich, Steinheim, Germany). The liposomes were subsequently eluted from the column by Na^+^ buffer (100 mM NaCl, 10 mM Mops/Tris pH 7.0). Immediately before an experiment, the eluate was further diluted 1/4 with the Na^+^ buffer. The fluorescence signals (Fig. 7A,B) were measured with a fluorescence microplate reader (NanoScan LF400, IOM, Berlin, Germany) or a fluorescence spectrophotometer (F-7000, HITACHI, Japan) (Fig. 7C). In the microplate experiments Fluo3 was excited at 488 nm and lucigenin at 456 nm. In both cases the emitted light passed through a 505 nm dichroic and a 520/10 band path filter. Fluo3 fluorescence measured in the fluorescence spectrophotometer was excited at 506±5 nm and at an emission wavelength of 526±5 nm.

### MD simulation

The system was constructed using VMD [32] and described by with the CHARMM22 potential function for proteins [33], CHARMM27 for phospholipids [34], the TIP3P water model [35], and ion parameters developed by Roux [36]. All titratable residues were kept at their standard protonation states. The simulation has been performed with a modified version of the program NAMD 2.6 [37]. Starting with the experimental NMR structure [13], the protein was inserted into a pre-equilibrated POPC membrane by removing overlapping lipid molecules, leading to 26 and 28 POPC molecules in each layer. The system was solvated by ca. 150 mM aqueous KCl solution (15 K^+^ and 6 Cl^-^ ions per 4050 water molecules), the positive net charge of the protein was therefore balanced by counterions. The total system contained 19641 atoms. The simulation time step was 2 fs, electrostatics were treated by the particle mesh Ewald method [38] on a 64x64x128 grid. Nonbonded real-space interactions were smoothly switched off over a range of 10–12 Å. Bond distances to hydrogen atoms were kept fixed. Production simulations were performed in the isothermal-isobaric (*NpT*) ensemble using the Langevin piston algorithm at 1 atm [39], [40] with an oscillation period of 800 fs and damping constant of 400 fs, and the Langevin thermostat with a coupling constant of 5 ps^-1^. After minimization over 10000 steps we successively turned on the barostat and the thermostat over 0.182 ns, followed by 5 ns *NpT* simulation with fixed protein backbone and 10 ns *NpT* simulation with c.o.m. constraint applied to the protein. Data for the fully free system were collected over 180 ns. The helicity of the protein backbone was analyzed with the STRIDE algorithm [41].
